# The Presence of Cu Facilitates Adsorption of Tetracycline (TC) onto Water Hyacinth Roots

**DOI:** 10.3390/ijerph15091982

**Published:** 2018-09-11

**Authors:** Xin Lu, Beibei Tang, Qi Zhang, Lizhu Liu, Ruqin Fan, Zhenhua Zhang

**Affiliations:** 1 Institute of Agricultural Resource and Environmental Sciences, Jiangsu Academy of Agricultural Sciences, Nanjing 210014, China; luxin@jaas.ac.cn (X.L.); tangbeibei2018@163.com (B.T.); 18752782205@163.com (Q.Z.); liulizhu@jaas.ac.cn (L.L.); fanruqin2007@126.com (R.F.); 2 Institute of Environmental Science and Engineering, Yangzhou University, Yangzhou 225009, China; 3 School of Agriculture and Environment, The University of Western Australia, Perth, WA 6009, Australia

**Keywords:** adsorption, tetracycline (TC), copper (Cu), complexation, water hyacinth roots

## Abstract

Batch experiments were conducted to investigate the adsorption characteristics of tetracycline (TC), and the interactive effects of copper (Cu) on the adsorption of TC onto water hyacinth roots. TC removal efficiency by water hyacinth roots was ranging from 58.9% to 84.6%, for virgin TC, 1:1 TC-Cu and 1:2 TC-Cu. The Freundlich isotherm model and the pseudo-second-order kinetic model fitted the adsorption data well. Thermodynamics parameters *ΔG*^0^ for TC were more negative in the TC plus Cu than the TC-only treatments, indicating the spontaneity of TC adsorption increased with increasing of Cu concentrations. An elevated temperature was associated with increasing adsorption of TC by water hyacinth roots. The additions of Cu(II) significantly increased TC adsorption onto water hyacinth roots within the pH range 4 to 6, because copper formed a strong metal bridge between root surface and TC molecule, facilitating the adsorption of TC by roots. However, Cu(II) hindered TC adsorption onto water hyacinth roots on the whole at pH range from 6–10, since the stronger electrostatic repulsion and formation of CuOH^+^ and Cu(OH)_2_. Therefore, the interaction between Cu(II) and TC under different environmental conditions should be taken into account to understand the environmental behavior, fate, and ecotoxicity of TC.

## 1. Introduction 

Tetracycline (TC) has been widely incorporated into animal feeds to minimize diseases and promote livestock growth rates. However, residues of TC are frequently detected in water samples from swine and poultry farms because TC is poorly assimilated during metabolism: approximately 50–80% is excreted via feces and urine as the non-metabolized parent compound [1,2]. There is a growing concern about increased antibiotic (i.e., sulfonamide and tetracycline antibiotics) resistance genes among microorganisms due to the continuous introduction of the antibiotics into the ecosystem [2,3]. On the other hand, Cu is another feed additive employed in intensive farming to promote animal growth; hence, the concentration of Cu has been widely detected at a mg/kg level in animal manure and wastewater from swine and poultry farms [4].

In practice, combined pollution is common, with the simultaneous presence of TC and Cu frequently reported in soil and aquatic environments [4,5]. However, most studies so far have focused on the environmental dynamics, toxicity and removal of TC and Cu, individually or together [6,7,8]. By contrast, few studies have dealt with the combined pollution of TC and Cu, resulting in the poor understanding of environmental dynamics of TC or Cu in co-contaminated soil and aquatic systems. Tetracycline contains multiple O- and N-functional groups in the molecule that act as potential electron donors to coordinate metal ions, i.e., copper, zinc, and calcium via complexation [5,9]. The formation of complex between TC and metal could modify the adsorption and desorption [10,11,12], photolysis [13], and bioavailability [14] of TC and metal ions, which would influence their removal efficiency.

Although water hyacinth has been considered as a potential plant to remove heavy metals and antibiotics from polluted water [5,15], few studies characterized the removal mechanisms of heavy metals and antibiotics by water hyacinth. During phytoremediation, the fibrous roots of macrophytes, dangling underwater in a dense mat, can effectively contact and trap the pollutants, then sorb assimilate and degrade them through the complex root systems. Therefore, root adsorption should be considered a vital process in removing pollutants via phytoremediation. However, the knowledges of the antibiotics/heavy metals adsorption onto the plant roots during phytoremediation is scarce. Additionally, the interactive effects of heavy metals and antibiotics on their adsorption by roots are still unknown, which might hinder the understanding of removal mechanisms and the improvement of the phytoremediation efficiency.

Water hyacinth possess a complex root system, with root length varying from 200 to 2000 mm and root surface area varying from 30 to 60 m^2^ per individual macrophyte [16]. The roots of water hyacinth may play an important role in the removal of heavy metals and antibiotics. Hence, main objectives of this study were to investigate the kinetic and isotherm characteristics of tetracycline adsorption onto water hyacinth roots as influenced by Cu. This study enhanced our understanding of the mechanism of removal tetracycline from wastewater to improve the removal efficiency of TC in the phytoremediation.

## 2. Materials and Methods

### 2.1. Chemicals and Solutions

Tetracycline hydrochloride (TC, 97.7% purity, CAS No. 64-75-5) was obtained from Dr. Ehrenstorfer Co. (Augsburg, Germany, chemical structures shown in Chen and Huang [13]). HPLC grade methanol was purchased from Tedia Company (Fairfield, OH, USA). Other chemicals such as KNO_3_, Ca (NO_3_)_2_, NaH_2_PO_4_, etc., were of analytical reagent grade and purchased from the Sinopharm Chemical Reagent Co., Ltd. (Shanghai, China). Deionized water (18 MΩ cm^−1^) was used for all the experiments.

### 2.2. Plant Collection, Acclimation and Preparation

Water hyacinth plants of uniform size and weight were sampled from an uncontaminated pond located on the campus of Jiangsu Academy of Agricultural Science (JAAS, 32°02′ S, 118°52′ E). Plants were washed thoroughly with tap water and planted in a plastic box (18 cm × 38 cm × 50 cm) containing half-strength Hoagland nutrient solution [17] with initial pH at 6.0 in the greenhouse. The solution was changed every 3 days. After acclimating for 2 weeks, the plants were harvested and washed with tap water followed by distilled water. The roots were separated and dried at 65 °C for 48 h. The dried roots were soaked in deionized water for several hours to ensure the weight of roots similar to their fresh weight. Finally, the excess water on the root surface was removed by filter paper and roots were cut into 0.5-cm-long segments prior to experiment.

### 2.3. The Point of Zero Charge (pH_PZC_) of Water Hyacinth Roots

The surface property of water hyacinth root could be characterized in terms of its point of zero charge (pH_PZC_). In the experiment, 0.5 g roots was added into a 50 mL PTFE centrifuge tube containing 25 mL half-strength Hoagland nutrient solution. The initial solution pH (pH_initial_) ranged from 2 to 10 after adjustment with 0.4 mM HCL or NaOH. The solutions with roots were shaken for 24 h to reach equilibrium. This was followed by measuring the pH (pH_final_). Then the pH_final_ vs. pH_initial_ were plotted for each set of samples, and a plateau in the pH final value was noted as the point of zero charge (pH_PZC_) of roots. 

### 2.4. The Complexation of TC with Cu(II)

A stock solution of tetracycline (10 mM) was prepared by dissolving tetracycline hydrochloride in methanol and was stored at 4 °C in the dark. A stock solution of copper (10 mM) was made by dissolving CuSO_4_·5H_2_O in deionized-water. The continuous variation, namely Job’s method was employed to determine the stoichiometry and stability constant of certain metal-ligand complex. Differences in the UV spectrum of the metal ion-ligand complex in comparison to the spectrum of the free metal ion plus free ligand was investigated [18]. In this study, a series of solutions in which the sum of total Cu(II) and TC concentration remained the same (100µM) but their concentration ratio varied. Different stock solutions of Cu(II) and TC were added into the 100 mL brown-glass volumetric flasks by adding half-strength Hoagland nutrient solution (pH = 6.0) to the mark. Additionally, mole ratio method was also employed. A fixed TC concentration of 20 µM with varying Cu concentrations at 0, 5, 10, 20, 50, 100 and 200 μM respectively were prepared by adding appropriate volumes of stock solutions into the 100 mL brown-glass volumetric flasks and adding half-strength Hoagland nutrient solution (pH = 6.0) to the mark. All the mixtures were shaken for 3 h at a speed of 180 rpm, 20 ± 0.5 °C in the dark. Then the mixtures were stood 1 h to ensure the formation of complexes between TC and Cu(II). The type of TC-Cu complexes were determined by ultraviolet-visible (UV/VIS) absorption spectra in the range of 250–450 nm (UV-1810 spectrophotometer, Puxi, Beijing, China). The wavelength resolution (Δλ) was 1 nm.

### 2.5. Batch Adsorption Experiment.

The adsorption of TC onto water hyacinth roots with or without Cu(II) was evaluated using batch experiments according to the Organization for Economic co-operation and Development (OECD) guidelines [19]. All adsorption experiments were performed in 50 mL PTFE centrifuge tubes.

#### 2.5.1. Adsorption Kinetics of TC in the Presence/Absence of Cu

According to the complexation experiment (Section 2.4 above), two complexes between TC and Cu, i.e., 1:1 TC-Cu (mole ratio) and 1:2 TC-Cu were observed and confirmed to be the relatively typical and stable species. Therefore, in the following experiment, 0.5 g water hyacinth roots were added to the PTFE centrifuge tubes followed by addition of 25 mL of either 50 µM TC, 1:1 TC-Cu or 1:2 TC-Cu solutions. The 1:1 TC-Cu and 1:2 TC-Cu complexes were prepared as the method described in Section 2.4. Then the mixtures were shaken at 180 rpm and 20 °C on the shaker (HZQ-F160, Jiangsu Changzhou, China) in the dark and sampled at time intervals of 0.25, 0.5, 1, 2, 4, 8, 16, 24, 48, 72 and 120 h. The suspensions were filtered through 0.22 µm glass fiber membrane and aliquots of the filtrate were analyzed for TC by HPLC. The control treatment did not contain water hyacinth roots. All the treatments were performed in triplicates and the average values were presented. The amount of TC absorbed by roots was calculated as the difference between the TC amount in solution at time = 0 and the various sampling points.

#### 2.5.2. Adsorption Isotherm of TC in the Presence/Absence of Cu

The batch experiments were carried out by adding 0.5 g of water hyacinth roots into 25 mL of TC solutions with concentration at 0, 10, 20, 50, 100, 200 µM respectively in the absence (0 µM) or presence of Cu (50 or 100 µM) at three temperatures (283, 293 and 303 K). The control treatments did not contain roots. The samples were taken and analyzed as described above. All experiments including the controls were conducted in triplicate. The solution pH was determined before and after adsorption equilibrium. The thermodynamic parameters including *ΔG*^0^, *ΔH*^0^ and *ΔS*^0^ were calculated.

#### 2.5.3. Adsorption of TC as Affected by pH and Cu(II)

The effects of pH on TC adsorption on water hyacinth roots as affected by Cu(II) were investigated as follows: 25 mL of half-strength Hoagland nutrient solutions containing TC (initial concentration: 50 µM) and Cu (0, 50 or 100 µM) was added to 50 mL PTFE centrifuge tubes. After shaking for 3 h to allow fully contact between TC and Cu, 0.5 g of water hyacinth roots were added. The pH of suspension was adjusted to the different values between 2 and 10 with HCl or NaOH. The solution pH was determined before and after adsorption equilibrium. The amount of absorbed TC was calculated by the difference between original and equilibrium concentrations.

### 2.6. Analysis of TC 

The concentration of TC in the supernatant filtrate was analyzed directly using an Agilent™ 1260 Series high performance liquid chromatography (HPLC) system (Agilent, Santa Clara, CA, USA) equipped with DAD operated at wavelength of 280 nm and an ZORBAX 300SB-C18 column (4.6 × 150 mm, 5 μm). Mobile phase was composed of 0.01 M oxalic acid (pH 4.0), methanol and acetonitrile (66.6:20:13.4, *v*/*v*/*v*) at a flow rate of 0.3 mL min^−1^. The column oven temperature was set at 30 °C, and the injection volume was 20 μL. The calibration curve was significantly linear in the concentration range of 2-100 µM (*p* < 0.05, *r*^2^ = 0.9993).

### 2.7. Data Analysis

The amount of TC adsorbed to the roots of water hyacinth was calculated by subtracting the value obtained from the initial concentration and the equilibrium concentration of TC in different treatments by using the equation [20]:

(1) $$C_{s}={(C}_{0}-C_{e})\times M\times V_{s}/m\;$$

where *C_s_* is the amount adsorbed (mg/g) by roots, *C_e_* is the equilibrium solution-phase concentration (µM), *C*_0_ is the initial concentration of TC (µM), M is mole mass (g/moL), *V_s_* is the solution volume (L), *m* is the weight of roots (g).

To further understand the kinetic characteristics, these data were fitted by the pseudo-first-order and pseudo-second-order kinetic models.

The pseudo-first-order kinetic:

(2) $$\ln(q_{e}-q_{t})=\ln q_{e}-k_{1}t\;$$

or the pseudo-second-order kinetic model:

(3) $$\frac{t}{q_{t}}=\frac{t}{q_{e}}+\frac{1}{k_{2}q_{e}^{2}}\;$$

where *k*_1_ (h^−1^) and *k*_2_ (g mg^−1^ h^−1^) are the adsorption rate constant of pseudo-first-order and pseudo-second-order kinetic model, respectively, *q_e_* (mg g^−1^) is the amount of TC sorbed on the adsorbent at equilibrium time, and *q_t_* (mg g^−1^) is the amount of TC sorbed on the adsorbent at any time (*t*).

Equilibrium isotherms are widely used to represent the relationship between the sorbed concentration on the adsorbent phase and the dissolved concentration at equilibrium. The adsorption isotherm data over the range of TC concentrations were estimated using the linear model:

(4) $$q_{e}=A+BC_{e}\;$$

or the Freundlich equation:

(5) $$\ln q_{e}=\ln k_{f}+\frac{1}{n}\ln C_{e}\;$$

or the Langmuir model:

(6) $$\frac{C_{e}}{q_{e}}=\frac{C_{e}}{q_{\max}}+\frac{1}{bq_{\max}}\;$$

where *q_e_* is the amount of TC sorbed (mg g^−1^); *C_e_* is the equilibrium concentration of TC (mg L^−1^); *k_f_* (mg g^−1^) (mg^−1^ L)^1/n^ is the Freundlich adsorption coefficient (estimating adsorption capacity), and 1/n is the Freundlich exponent (estimating the adsorption intensity). When 0 < 1/*n* < 1, the adsorption is favorable; when 1/*n* = 1, the adsorption is irreversible; and when 1/*n* > 1, the adsorption is unfavorable [21]. In the Langmuir model, *q_max_* is the maximum adsorption capacity (mg g^−1^), and b is the adsorption affinity constant related to the binding energy of adsorption.

The adsorption of TC with or without Cu on water hyacinth roots was studied at 283, 293 and 303 K. The equilibrium constant (*K_C_*) was determined by using the following equation:

(7) $$K_{C}=\frac{C_{A}}{C_{S}}\;$$

where *C_A_* and *C_S_* are the concentrations of TC sorbed onto water hyacinth roots and in solution at equilibrium respectively. The change of standard molar Gibbs free energy was calculated based on the Gibbs equation:

(8) $$\Delta G^{0}=-RTlnK_{C}\;$$

where *R* is the universal gas constant (8.314 J mol^−1^ K^−1^) and *T* is absolute temperature (*K*). The thermodynamic parameters like *ΔH*^0^ and *ΔS*^0^ were calculated by using the Van’t Hoff equation and plotting a graph of *lnKc* vs 1/*T*, where slope gave the value of *ΔH*^0^ and intercept represented *ΔS*^0^:

(9) $$lnK_{C}=\frac{\Delta S^{0}}{R}-\frac{\Delta H^{0}}{RT}\;$$

Significant difference tests at p = 0.05 were performed by analysis of variance (ANOVA) using SPSS 18.0 (SPSS Inc., Chicago, IL, USA).

## 3. Results and Discussion

### 3.1. The Point of Zero Charge (pH_PZC_) of Water Hyacinth Roots 

pH_PZC_ is the pH in solution at which the investigated surface is at net zero charge. It is a surface intensive property that accounts for the proton transfer to and from the surface hydroxyl groups which causes dramatic pH shifts in an aqueous solution [22].

**Figure 1:**
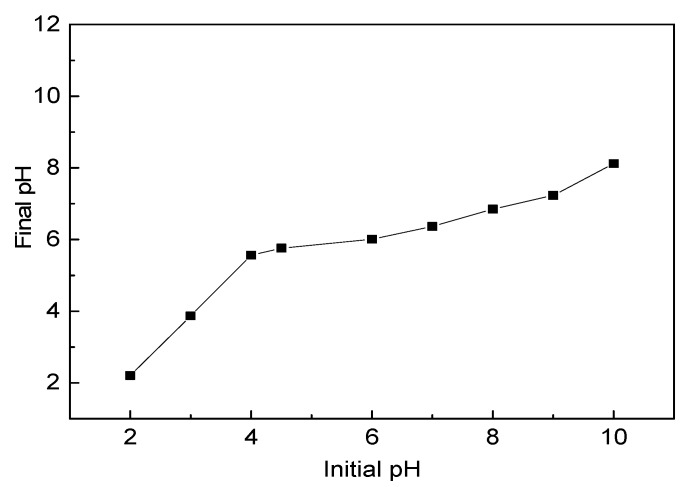
pH_PZC_ of water hyacinth roots.

Hence pH_PZC_ can be regarded as a simple and strictly surface sensitive method to measure surface composition [23], which can explain the correlations of reactivity between TC and water hyacinth roots. As seen in Figure 1, the pH_PZC_ of water hyacinth roots was found to be approximately 6.00, which indicated that the surface of the roots is positively charged at pH < 6.00 and negatively charged at pH > 6.00. In addition, it also indicated that the root system of water hyacinth had a strong buffer capacity, with initial pH value of solution changing from 2 to 10, the final pH value maintaining a relatively stable condition, especially at initial pH value > 4.00. 

### 3.2. Complexes of TC with Cu(II)

**Figure 2:**
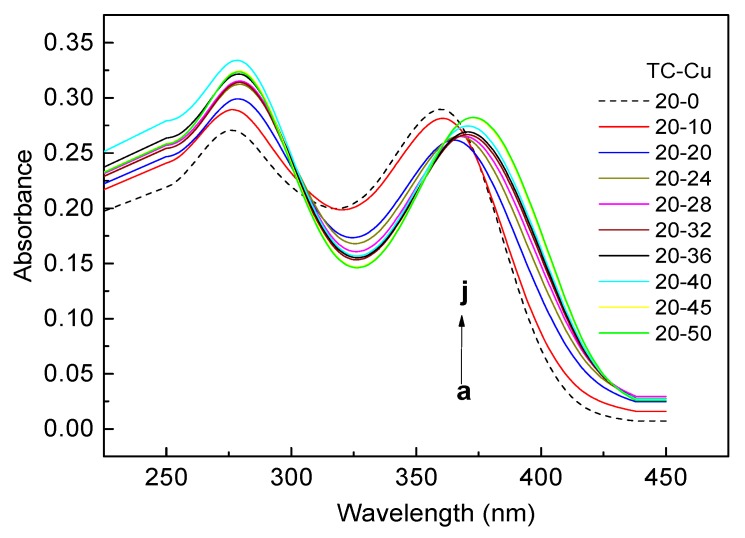
UV-Vis absorbance spectra of TC in the absence (dashed line) and presence (solid line) of Cu(II) ranging from 250 to 450 nm at [TC] = 2 0 µM with [Cu]^2+^ at: a, 0; b, 10; c, 20; d, 24; e, 28; f, 32; g, 36; h, 40; i, 45; j, 50 respectively (pH = 6).

The absorbance spectra of TC in virgin style (dashed line) showed two main bands and peaked at 275 and 360 nm, respectively (Figure 2). According to the previous study, π–π* transitions associated with the tricarbonylamide system (C1-C3) contribute only to the band near 275 nm, while π–π* transitions of the phenolic β-diketone system (C10-C12) contribute to both the 275 nm and 360 nm bands [24]. When TC cooperated with Cu, both of the absorbance band in 275 and 360 nm was observed to be markedly shifted from 275 to 279 nm and from 360 nm to 373 nm, respectively (solid line from a to j). In addition, the shift was more evident with an increase of the Cu concentrations in the coexistent Cu with TC, as a consequence of ligand coordination [25]. It was indicated that Cu(II) would readily react with TC and form yellow water-soluble complexes, which was predominated by the zwitterionic and positive charged complex at pH 6.0.

**Figure 3:**
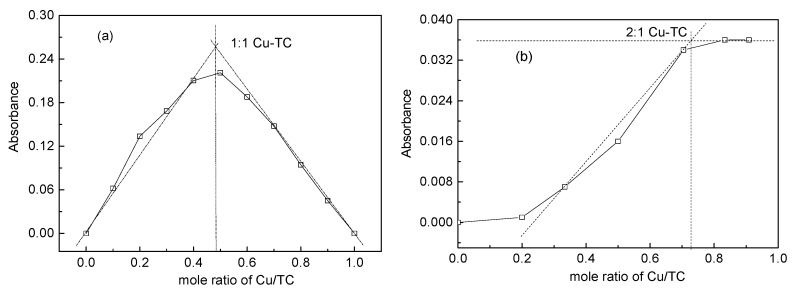
Complexation of Cu and TC: (**a**) 1:1 and (**b**) 2:1 complex at pH 6.0 by using Job’s method and Mole ratio method.

Job’s method [18] involves measuring the absorbance of a series of solutions in which the total ligand concentration and the total metal ion concentration (Cu) are varied, but their sum keep constant. The absorbance was determined and plotted against the ratio of Cu /TC (Figure 3a). Maximum absorbance occurred when Cu = TC (mole ratio is 0.5), indicating that 1:1 Cu-TC complex is predominant. Mole ratio method was also employed by measuring the absorbance of a series of solutions in which a fixed TC concentration mixed with varying Cu concentrations respectively. By plotting Cu concentration versus complex absorbance, and the complex formation constant could be determined from the slope and the x-axis intercept. The result showed that maximum absorbance occurred when the Cu concentration is close to 0.7, indicating that 2:1 Cu-TC complex is predominant. It is known that Cu(II) could chelate with TC when encountered in the aqueous solution. The complexation reaction between TC and Cu(II) results in different specific complex species, depending on the ratios of Cu to TC, mainly the concentration levels of metals. According to results obtained by Job’s method and mole ratio method [18,26], Cu at low levels, 1:1 complex was formed. A further increase in Cu level lead to the production of 2:1 complex (Figure 3), which was in agreement with the results previously reported by Wen et al. [27] and Zhao et al. [28].

### 3.3. Adsorption Kinetics Experiment

**Figure 4:**
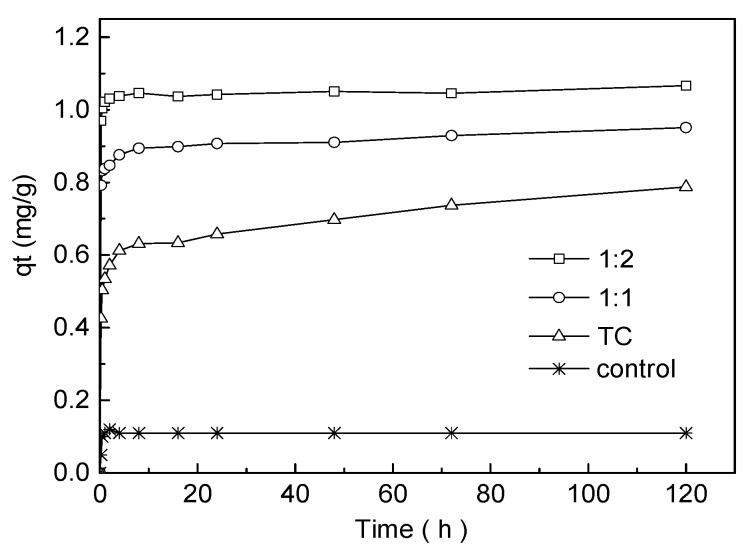
Adsorption kinetics of TC by water hyacinth roots in the absence or presence of Cu(II). Experimental conditions: [TC] = 50 µM, [Cu^2+^] = 0, 50 and 100 µM, respectively.

The adsorption kinetics of TC in the absence or presence of Cu(II) onto water hyacinth roots are shown in Figure 4. The adsorption of TC can be divided into a two-stage reaction involving a rapid and slow adsorption process. During the first rapid adsorption stage, about 58.9%, 75.8%, 84.6% of TC was adsorbed within 24 h for virgin TC, 1:1 TC-Cu and 1:2 TC-Cu, respectively, which could be due to physical adsorption or ion adsorption on the surface of the absorbent [29]. Then, a slow adsorption stage was followed in which the adsorption rate drop toward a steady state after 24 h for both 1:1 TC-Cu and 1:2 TC-Cu complex. There was no significant difference (*p* < 0.05) in the adsorption amount from 24 h to 120 h between 1:1 TC-Cu and 1:2 TC-Cu complex. By comparison, the amount of virgin TC adsorbed by roots continuously increased at a slow rate after 24 h, indicating the adsorption of TC onto water hyacinth root was dominated by microprecipitation and surface complexation between the root surface functional groups and TC through a physical adsorption mechanism. Compared with virgin TC, 1:1 TC-Cu and 1:2 TC-Cu complex could provide metal bridges and coordinate strongly with root surface functional groups to form binary and ternary complexes, which might be a fast process thus rapidly going on and reaching the equilibrium [30]. Under comprehensive consideration, 24 h was taken as the equilibration time for TC adsorption on water hyacinth roots in the following adsorption experiments.

**Figure 5:**
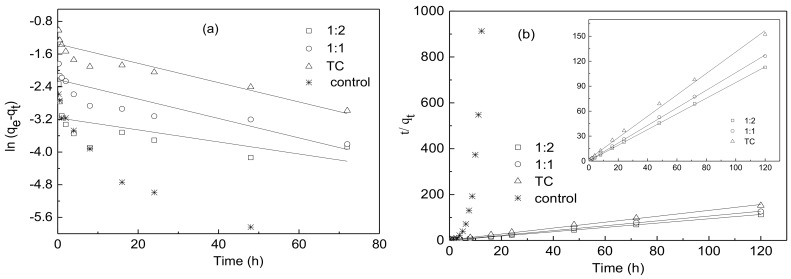
Adsorption kinetic curves of TC with or without Cu(II) onto water hyacinth roots fitted by (**a**) pseudo-first-order kinetic model (**b**) pseudo-second-order kinetic model Dots: measured data, solid line: curve fitted into the models.

Adsorption kinetics of TC on water hyacinth roots fitted well the pseudo-second-order kinetic model (R^2^ = 0.996, 0.999 and 0.999) rather than the pseudo-first-order kinetic model (R^2^ = 0.844, 0.768 and 0.346), respectively, for TC, 1:1 TC-Cu and 1:2 TC-Cu (Figure 5 and Table 1). The pseudo-second-order kinetics model has been widely applied to describe the chemisorption of pollutants from aqueous solutions involving valency forces through sharing or exchange of electrons between the adsorbent and adsorbate, as covalent forces and ion exchange [29,31]. By fitting the data into the linearized form of the pseudo-second-order kinetics model, the amounts of TC adsorbed on water hyacinth roots at equilibrium (*q_e_*) were 787, 951 and 1067 mg kg^−1^ for TC, 1:1 TC-Cu and 1:2 TC-Cu, respectively, which was quite similar to that reported for biochar and much higher than that observed for cinnamon soil and red soil [29,32]. According to the pseudo-second-order kinetic model, both the parameters *k*_2_ and *q_e_* increased with the presence concentrations of Cu (Table 1), which indicated that the coexistence of Cu could enhance the adsorption rate and amount of TC onto water hyacinth roots. The main reason could be attributed to the strong chelating capability of TC and Cu(II), in which Cu played as a bridge across the root surface and TC. In addition, the increasing ratios of Cu to TC associated with an increase of TC adsorption amount on roots, which indicated that metal bridging was one of the most important adsorption mechanisms of TC on water hyacinth roots. Therefore, a stronger bridge between root surface and TC was formed across Cu for 1:2 than 1:1 TC-Cu complexes, due to more Cu available for the bridging between TC and root surface. Other researchers have got the similar results about the co-sorption of TCs (including TC, OTC and CTC) and Cu onto soils [12], and modified mesoporous silica [33]. The lower adsorption rate of TC onto loess soil in the presence of Cu compared with TC alone observed by Liu and Wu [34] was attributed to the fact that the complexation between TC and Cu would enlarge the size of the contaminants and slow down the internal mass transfers in the adsorbent.

### 3.4. Adsorption Isotherm Experiment

**Figure 6:**
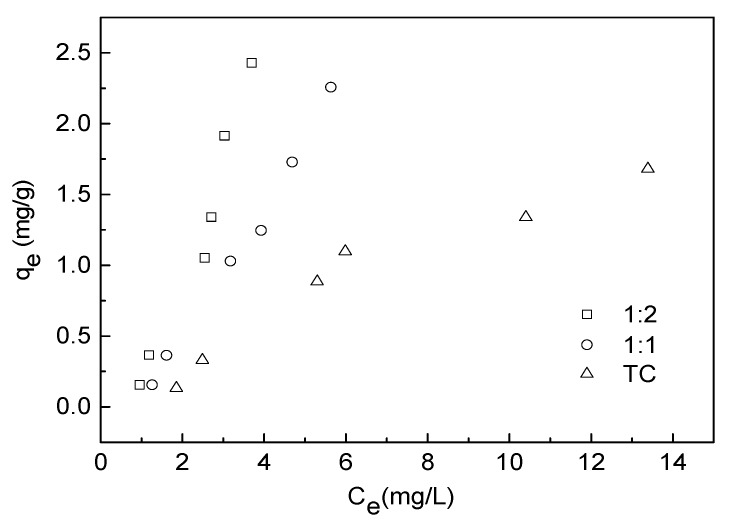
Adsorption isotherms of different TC-Cu complexs onto water hyacinth roots.

Adsorption isotherms of TC on water hyacinth roots in the absence and the presence of Cu are illustrated in Figure 6. The amounts of TC adsorbed increased with the increase of initial TC concentrations in the solution. In addition, an increase in TC adsorption on water hyacinth roots associated with the increasing concentration of Cu in cooperation.

**Figure 7:**
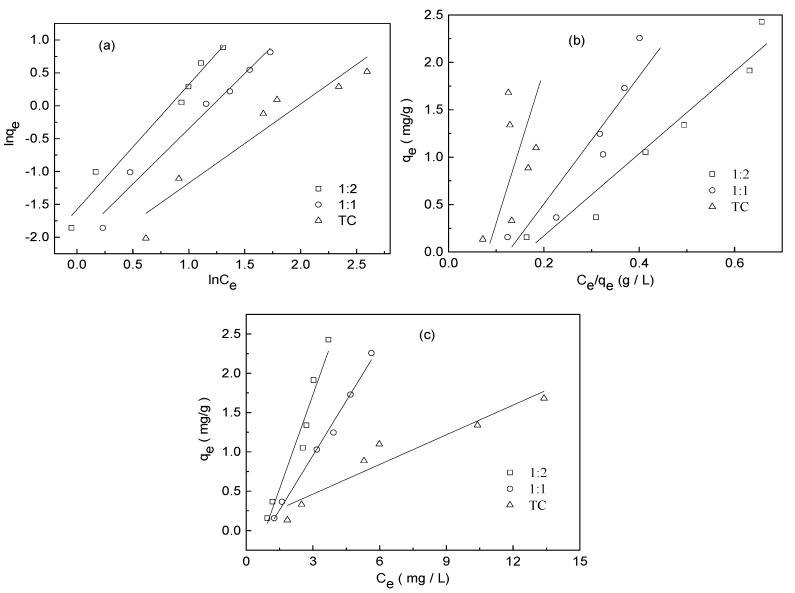
Three isotherm model plots for adsorption of TC in different complexation: (**a**) Freundlich, (**b**) Langmuir, and (**c**) Linear model (root dosage = 0.5 g/L, initial pH = 6.0 and equilibration time = 24 h).

Adsorption data of TC were fitted by three adsorption isotherm models as seen in Figure 7. The parameters and R^2^ values estimated by the three models are illustrated in Table 2. Poor performance of Langmuir was found with negative q_max_ values and relatively low correlation coefficient (R^2^) as seen in Table 2. By contrast, the adsorption data over the range of TC concentrations studied here could be well described by the Freundlich equation with R^2^ ranging from 0.892 to 0.973, which suggested that adsorption has taken place on a heterogeneous surface, and the uptakes of TC and Cu(II) on the adsorbent were multilayer adsorption. The Freundlich K_f_ values varied from 0.0926 to 0.2062 under different concentrations of Cu indicated that Cu(II) would increase the adsorption capacity of TC onto water hyacinth roots. The Freundlich exponents n of different states TC sorbed onto water hyacinth roots were between 0 and 1, which suggested that the higher TC concentrations has disadvantage for adsorption of additional molecules onto water hyacinth roots causing by the saturation of specific adsorption point and the decrease in adsorption affinity of residual adsorption point [1]. Similar results have been observed in clay minerals [35] The linear model was also used to model experimental data with high performance, which indicated the adsorption of TC by adsorbents was in linearity of isotherms at low adsorbate concentrations [36].

### 3.5. Adsorption Thermodynamic Parameters of TC in Different Complexation

**Figure 8:**
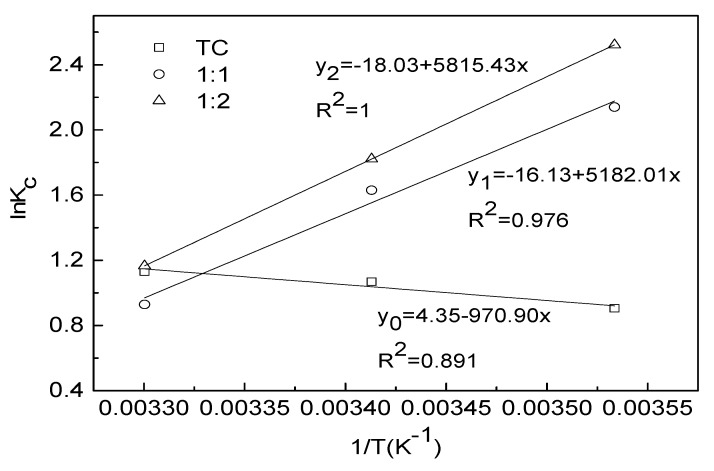
Plots of *lnKc* versus 1/*T* for calculating the activation energy of TC adsorption onto water hyacinth roots.

In-depth information regarding the inherent energy and structural changes of adsorbent after adsorption and the mechanism involved in adsorption process can be assessed properly though thermodynamics experiment [37]. As seen in Figure 8, the correlation coefficient between *lnKc* and 1/*T* were 0.891, 0.9767 and 1 for TC, 1:1 TC-Cu and 1:2 TC-Cu, respectively. The value of thermodynamics parameters were presented in Table 3. The positive value of *ΔH*^0^ (8.072 KJ mol^−1^) and *ΔS*^0^ (36.18 J (mol·K)^−1^) for virgin TC indicated that the adsorption of TC alone was endothermic with the adsorption capacity increasing as temperature increased. The entropy is positive because the *ΔS*^0^ increase ascribed by water molecules desorption is far greater than the entropy decrease due to tetracycline adsorption. In addition, the *ΔG*^0^ was found to be more negative with the temperature increasing from 283 K to 303 K (Table 3), indicating the increasing of reversibility and stability for TC adsorption onto water hyacinth roots.

By contrast, for the adsorption of TC in TC-Cu complexation by water hyacinth roots, both the values of *ΔH*^0^ and *ΔS*^0^ were found to be negative, indicated that the adsorption process was exothermic and the increase in temperature associated with the decrease in randomness at the solid/solution interface. Therefore, the adsorption of TC in TC-Cu complexation on water hyacinth roots was favored by lower temperature owing to its exothermic nature. The *ΔG*^0^ for TC in 1:2 TC-Cu complex was more negative than 1:1 TC-Cu complex, followed by TC alone, indicating the spontaneity and irreversibility of TC adsorption increased with Cu dose increased.

The *ΔG*^0^ values illustrated in Table 3 were within the ranges of −20 and 40 KJ mol^−1^, which indicated that the physical adsorption was the dominant mechanism [38]. However, according to the pseudo-second-order kinetic model, the adsorption of TC onto water hyacinth roots was controlled by the chemical adsorption regardless of Cu co-existence. Hence, it could be speculated that there were both physical and chemical adsorption mechanism working during the adsorption process of TC onto water hyacinth roots regardless of Cu additions. Putra et al. [39] also indicated that both physisorption and chemisorption played important roles for the adsorption process of amoxicillin from wastewater by activated carbon.

### 3.6. Sorption of TC onto Water Hyacinth as Affected by pH and Cu(II)

**Figure 9:**
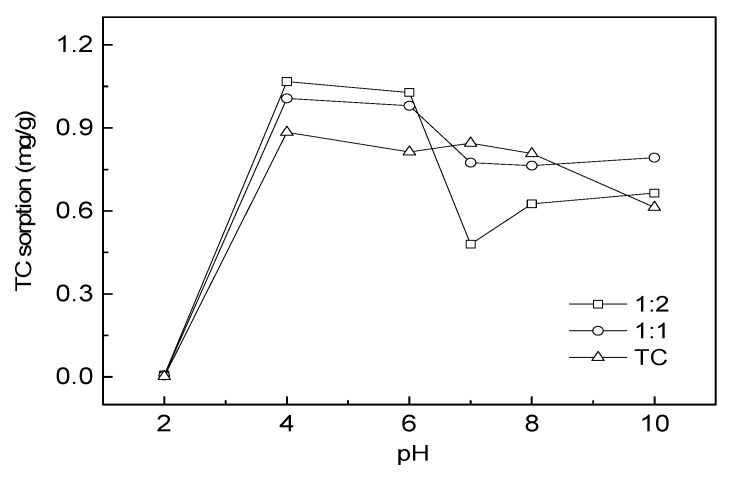
Effect of pH on TC adsorption onto water hyacinth root in the presence or absence of Cu.

The effect of pH on the adsorption of TC onto water hyacinth roots in the absence and presence of Cu was shown in Figure 9. It is found that pH was important factor affecting the TC adsorption process onto water hyacinth roots, because the charge of the adsorbent surface and the TC species presented in the solution were both dependent on the pH.

A previous study indicated that an increase of TC adsorption capacity was observed with increasing pH for tetracycline under low acidity conditions [40]. Furthermore, low-acid and high-alkaline solution can accelerate the adsorption of TC onto rice husk ash [41], soil and sediment [7]. When solution pH was over 6.0, TC adsorption onto water hyacinth roots became decline in the absence of Cu(II). At pH = 2.0, the TC molecule was protonated (H_3_L^+^), the surface of water hyacinth was positively charged; hence, the TC adsorption was reduced by the electrostatic repulsion between the H_3_L^+^ and the adsorbent surface. In the range of pH 4.0 to pH 6.0, the TC molecule was neutral, while the root surface of water hyacinth (pH_pzc_ = 6.0) was in positive charge. Therefore, the lowest electrostatic repulsion was experienced between TC molecules and the surface of the roots in this pH range. When pH further increased, the cationic TC molecule changes to the zwitterion species H_2_L or anionic species like HL^−^ and L^2−^, and the surface of water hyacinth root was negatively charged. Thus the adsorption affinity of TC to water hyacinth root was reduced due to the enhancement of electrostatic repulsion. Zhao et al. [28] found that pH greatly influenced TC adsorption onto goethite with the maximal adsorption at pH 8.5, while the adsorption was beginning to decrease due to the electrostatic repulsion with further increase of the pH.

The presence of Cu significantly promoted the adsorption of amounts of TC onto roots over the pH ranging from 2.0 to 6.0, while TC adsorption was reduced when the pH was between 6.0 and 10.0. It was possible that Cu^2+^ could complex with TC (H_3_L^+^, H_2_L, and HL^−^) to form CuH_2_L^2+^, CuHL^+^, and CuL species, which would help to decrease the negatively charged moieties in TC molecules, and enhance the TC adsorption onto water hyacinth [7]. However, Cu(II) hindered TC adsorption onto water hyacinth roots on the whole at the pH range from 6–10, due to the stronger electrostatic repulsion and the formation of CuOH^+^ and Cu(OH)_2_ with increasing pH [42]. The results indicated that the interactions between Cu(II) and TC at the different pH values should be taken into account to understand the environmental behavior, fate, and ecotoxicity of TC, which are associated with its removal efficiency.

## 4. Conclusions

Spectroscopic measurements showed that Cu(II) could complex with TC to form 1:1 or 1:2 complexes, depending on the concentration ratios between TC and Cu. Adsorption of TC onto water hyacinth roots in the presence or absence of Cu(II) could be established in 24 h. The pseudo-second-order kinetic model and the Freundlich model were found to be suitable to characterize the adsorption process of TC onto water hyacinth roots. Adsorption capacity of TC onto water hyacinth roots were influenced by the presence of Cu(II) and the pH value. The presence of Cu significantly promoted TC adsorption onto roots by 13.8–26.4% over the pH range from 2.0 to 6.0, possibly due to metal complexation and surface-bridging mechanisms. However, TC adsorption was hindered by 5.4–43.3% in the presence of Cu(II) under alkaline conditions, due to the stronger electrostatic repulsion and the formation of CuOH^+^ and Cu(OH)_2_ with increasing pH. The change of standard molar Gibbs free energy (*ΔG*^0^) was found to be negative for TC regardless of Cu addition, which indicated the spontaneity of TC adsorption by water hyacinth roots. The *ΔG*^0^ for TC in 1:2 TC-Cu complexations was more negative than for 1:1 TC-Cu complexation, followed by TC alone, indicating the spontaneity and irreversibility of TC adsorption increased as the Cu dose increased. To understand the removal mechanisms and promote the remediation efficiency of antibiotics during phytoremediation, further researches should be focused on the absorption mechanism of antibiotics into plant roots, the translocation and accumulation mechanism of antibiotics in the different parts of plant tissues.

## Figures and Tables

**Table 1:** Kinetic parameters of the pseudo-first-order and pseudo-second-order kinetic models for adsorption of TC onto the roots of water hyacinth with or without Cu(II).

Condition	*q*_e,exp_ (mg g^−1^)	Pseudo-First-Order Kinetic Model			Pseudo-Second-Order Kinetic Model		
		*q*_e,cal_ (mg g^−1^)	*k*_1_ (h^−1^)	R^2^	*q*_e,cal_ (mg g^−1^)	*k*_2_ (mg g^−1^ h^−1^)	R^2^
TC	0.78745	0.25855	0.02367	0.844	0.77737	0.69354	0.996
1:1	0.95123	0.10802	0.02379	0.768	0.94619	2.03175	0.999
1:2	1.06662	0.04234	0.01476	0.346	1.06207	4.41873	0.999

**Table 2:** Adsorption isotherm parameters for TC onto water hyacinth roots in the absence or the presence of Cu.

Adsorbate	Freundlich Model			Langmuir Model			Linear Model		
	K_f_ ((mg g^−1^) (mg^−1^ L)^1/n^)	n	R^2^	*q_max_* (mg/g)	b	R^2^	A	B	R^2^
TC	0.0926	0.8313	0.892	0.0548	−0.1573	0.510	0.0838	0.1260	0.903
1:1 TC−Cu	0.1314	0.5955	0.973	−0.1421	−0.2190	0.776	−0.4349	0.4629	0.989
1:2 TC−Cu	0.2062	0.5261	0.970	−0.3784	−0.2717	0.927	−0.6676	0.7976	0.941

**Table 3:** Thermodynamics parameters for TC adsorption onto water hyacinth.

Condition	*ΔH*^0^/KJ·mol^−1^	*ΔS*^0^/J·(mol·K)^−1^	*ΔG*^0^/KJ·mol^−1^		
			283 K	293 K	303 K
TC	8.072	36.18	−2.130	−2.604	−2.848
1:1	−43.08	−134.13	−5.035	−3.970	−2.340
1:2	−48.35	−149.88	−5.933	−4.439	−2.935
